# Methodology for Calculating the Damaged Surface and Its Relationship with Power Loss in Photovoltaic Modules by Electroluminescence Inspection for Corrective Maintenance

**DOI:** 10.3390/s24051479

**Published:** 2024-02-24

**Authors:** Nieves Saborido-Barba, Carmen García-López, José Antonio Clavijo-Blanco, Rafael Jiménez-Castañeda, Germán Álvarez-Tey

**Affiliations:** Departamento de Ingeniería Eléctrica, Universidad de Cádiz, Avenida de la Universidad de Cádiz 10, 11519 Puerto Real, Cádiz, Spain; carmen.garcia@uca.es (C.G.-L.); joseantonio.clavijo@uca.es (J.A.C.-B.); rafael.castaneda@uca.es (R.J.-C.); german.alvarez@uca.es (G.Á.-T.)

**Keywords:** photovoltaic modules, electroluminescence, defects, substring, defect analysis tool, performance

## Abstract

Photovoltaic panels are exposed to various external factors that can cause damage, with the formation of cracks in the photovoltaic cells being one of the most recurrent issues affecting their production capacity. Electroluminescence (EL) tests are employed to detect these cracks. In this study, a methodology developed according to the IEC TS 60904-13 standard is presented, allowing for the calculation of the percentage of type C cracks in a PV panel and subsequently estimating the associated power loss. To validate the methodology, it was applied to a polycrystalline silicon module subjected to incremental damage through multiple impacts on its rear surface. After each impact, electroluminescence images and I-V curves were obtained and used to verify power loss estimates. More accurate estimates were achieved by assessing cracks at the PV cell level rather than by substring or considering the entire module. In this context, cell-level analysis becomes indispensable, as the most damaged cell significantly influences the performance of the photovoltaic model. Subsequently, the developed methodology was applied to evaluate the conditions of four photovoltaic panels that had been in operation, exemplifying its application in maintenance tasks. The results assisted in decision making regarding whether to replace or continue using the panels.

## 1. Introduction

Among renewable energies, solar photovoltaic (PV) energy is the cleanest. It is abundantly available, can be used for the maximum number of days in a year, and has low operating/maintenance costs for its implementation [1].

Thus, solar PV energy has become an important asset within renewable energies, not only because of its current contribution to electricity coverage but also because of its future growth prospects. According to the latest report of the International Energy Agency (IEA-PVPS), the demand for electricity covered by photovoltaics was 11.6% in 2022 compared to 8.2% in 2021 [2].

This increase in the installation of PV plants makes it necessary to develop control and preventive maintenance systems. Tools are needed to determine the state of PV modules by analyzing their degradation, since sometimes there is no possibility of seeing a failure except by means of visual inspection. Currently, power plants are growing bigger and occupying larger surfaces. As a result, it is becoming more and more necessary to analyze the condition of the modules on a massive scale, ideally without the need to disconnect them from the power plant. With the primary goal of executing more effective maintenance actions, there is a need for user-friendly tools that enable swift decision making. During the manufacturing of the PV modules, especially throughout the soldering processes, significant mechanical stresses are imparted onto the solar cells, leading to damage in the cells themselves, which could potentially impact production [3]. The damage produced during manufacturing has been the subject of study by various researchers over the last decade. For instance, the authors of [4] conducted a study on the damages incurred during the manufacturing process of monocrystalline silicon modules using an automated cell segmentation technique.

After the manufacturing process, PV modules can experience damage stemming from transport incidents, as well as mechanical impacts caused by wind and snow when they have been already installed, which result from adverse weather conditions [5]. In reference [6], a neural-network-driven fault detection algorithm was developed specifically for PV modules. This algorithm categorizes faults, speeding up fault detection. In [7], the occurrence of cracks was influenced by various factors, which could be categorized as environmental factors, installation factors, cost-related factors, and the interactions among them. In reference [8], Fourier’s theorem was employed to accurately and swiftly identify defects at the cell level.

Some studies have already been conducted on the origin of these cracks, their characteristics, and their influence on the energy performance of the modules [9]. The occurrence of cracks in PV modules can result in regions where individual cells become electrically disconnected from the rest, leading to a reduction in the panel’s output power [10,11]. In [12], an evaluation was conducted to estimate the power loss resulting from various PV failures, leading to the conclusion that power loss due to cell cracks can range from 1% to 15%. This decrease in power output has a substantial impact on the financial well-being of system owners [13,14].

Currently, several methods are available for assessing PV modules and enabling the detection of failures and the estimation of their degradation over time [15,16,17,18,19,20]. Some of these methods developed in [21], such as visual inspection, infrared thermography, I-V curve tracing, and electroluminescence (EL), have already been applied in a real 2.85 MW PV plant to detect failures, assess its operational status, and estimate its overall degradation [22]. In [23], the performance of c-Si modules in a desert environment was studied by visual inspections and IV curve field measurements.

Visual inspection is the most straightforward method for identifying anomalies around the PV panels. However, it may pose challenges in terms of achieving a comprehensive evaluation of the entire plant. I–V curve tracing is a well-established technique for evaluating plant degradation. Nevertheless, it may not detect small defects in the I-V characteristics [15] or pinpoint the exact location of the fault [24]. Moreover, these tests can be costly, labor-intensive, and time-consuming, especially when dealing with power plants covering vast surface areas.

A further step in the complexity, but also in the efficiency, of the method is represented by infrared thermography analysis (IRT) [25,26]. IRT is perhaps the most widely used technique for inspecting and determining PV module failures [16,17]. However, as indicated in reference [27], it is worth noting that certain hot spots identified in IRT may not necessarily be indicative of defects. In some cases, these thermal anomalies could be attributed to factors unrelated to module performance, such as environmental conditions or transient fluctuations. This method also has the limitation of being unable to detect specific types of damage to PV panels or, consequently, the faults inherent in them [28]. For instance, it may struggle to detect cracks in PV cells. In this context, cracks in PV panels may exhibit high temperatures, which can be detected through thermography. However, even if detected, it may not be possible to quantify the extent of the crack, which poses a challenge in estimating power loss [28].

The analysis of PV modules through EL testing has emerged as an alternative method for detecting anomalies related to cracks. In fact, this approach is increasingly gaining importance [18,19,20,29,30,31].

The analysis of PV modules using EL can be carried out manually, a process often considered expensive and time-consuming, additionally requiring a significant level of experience. Moreover, if this analysis were to be conducted through direct observation, different examiners might arrive at varying conclusions when examining the same resulting EL-image. This would imply comparisons among the different observations, potentially affecting the overall effectiveness of the test [32]. Thus, it is essential to establish a method capable of automatically analyzing the resulting EL-images and estimating their power loss. To achieve this, the automated methodology must be capable of accurately quantifying the area affected by the crack or damaged regions inside the panel, and then estimating the associated power loss.

The study of cracks in PV modules using EL image analysis techniques has been the focus of numerous publications. Reference [33] concludes that the effectiveness of these analyses depends on the quality of the EL image taken and which quality parameters of this image are the most influential. In reference [34], an electroluminescence-based crack analysis system, coupled with a cell fracture toughness tester, was proposed to gather information regarding cracked cells within a module. Research has also been conducted on how to perform diagnostic evaluations for damage resulting from cracking, particularly after the manufacturing, transportation, and installation of the modules [18]. The mechanical stability of the cells due to cell thickness has been studied [35], as well as the loss of power produced by PV modules due to the existence of microcracks [10].

New techniques for quantifying the area affected by a crack in a PV panel are currently being developed. In this context, automatic segmentation is essential to optimize the analysis of modules using EL. Within the automated segmentation process, the EL-image undergoes transformation into data, which are subsequently processed for statistical computation. This methodology facilitates the differentiation between damaged and undamaged areas within the module [36].

According to [37], cracks in a PV module can be categorized in three ways, designated as types A, B, and C. In general, cell microcracks, referred to as type A cracks, do not result in inactive cell areas and do not significantly reduce cell power. Type B cracks indicate partially disconnected zones, with the impact depending on the irradiance. Type C cracks represent areas that are completely electrically disconnected from the rest of the module. Type C cracks result in a severe loss of power, and, in some cases, can lead to module reverse polarization and the formation of hot spots.

The quantification of cracks and damaged areas in a photovoltaic module using the EL-image analysis method requires the determination of a threshold (TH) level [37]. This value divides the pixel-data into two zones, with each zone representing a group of tones. The TH level distinguishes between pixels classified as cracked areas and those classified as active areas.

Several procedures have been employed to determine this TH level. These procedures vary in sophistication, ranging from visual assessments to the utilization of image segmentation methods through thresholding for digital processing across various applications, such as in medicine or traffic [38]. Although most procedures typically incorporate image correction into their processes, these methods offer the advantage of effectively handling various anomalous situations, including noise and distortions, which may persist even after the correction, thereby enhancing the filtering of the EL image.

Various algorithms and methods have already been developed to identify Type C cracks and estimate the associated power loss [39,40,41,42]. In [43], models based on convolutional neural networks are proposed for the classification of defects or damage within the modules.

While various authors have developed interesting methods for estimating the cracked areas of PV panels, there is no common consensus regarding the power loss associated with the affected regions. Furthermore, in most publications, the relationship between cracked areas and power loss has been investigated for the entire panel without considering the internal connection of PV cells within the panel. In line with the authors’ understanding, as panels are typically divided into substrings based on the parallel connection of bypass diodes, the distribution of cracks across the panel holds significant relevance for the module’s power output.

In this paper, an EL image processing method based on the IEC TS 60904-13 specification is developed for the estimation of the proportion of type C cracks in PV modules. Then, with the proportion of type C cracks, the output peak power in STC is estimated.

Utilizing this method enables swift results to be obtained from an electroluminescence (EL) image of a photovoltaic panel when evaluating a considerable number of panels. In contrast to other authors seeking enhanced accuracy in their estimations, achieving notable results [40,42], the method presented in this paper prioritizes simplicity and practicality, albeit with a slight trade-off in power loss estimation accuracy. The proposed method’s approach facilitates a prompt response in decision making during corrective maintenance, aiding in the determination of whether a PV module should be replaced or retained. Thus, in contrast to other implemented methods, the primary contribution of the developed approach lies in its ease of application, a crucial factor in maintenance tasks; its rapid response; and its versatility in addressing various types of failures.

The method was applied to a new crystalline silicon module which was subjected to various impacts, resulting in an increased number of cracks. Each time the module received an impact, an EL-image was processed, and an I-V curve was recorded. Subsequently, the power loss measured in each instance was compared with the estimated power loss resulting from the cracked areas identified by the designed method. This study encompasses crack analysis and power loss evaluation across the entire module, by sub-module, and at the cell level, considering the PV panel’s internal electrical connections. The results demonstrate how cracks in a PV panel should be taken into account to obtain more accurate power loss estimations.

Subsequently, the developed methodology was used to evaluate the conditions of four photovoltaic panels in a solar power plant as an example of its application in maintenance tasks. The results guided decisions on whether to replace or continue using the panels. The paper is structured as follows: Materials and Methods provides a comprehensive description of the methodology employed for crack quantification and the study of performance. Then, in Results, the cracked areas are compared alongside the corresponding I-V curves collected in each case. Finally, the discussion of the research is presented.

## 2. Materials and Methods

**Figure 1:**
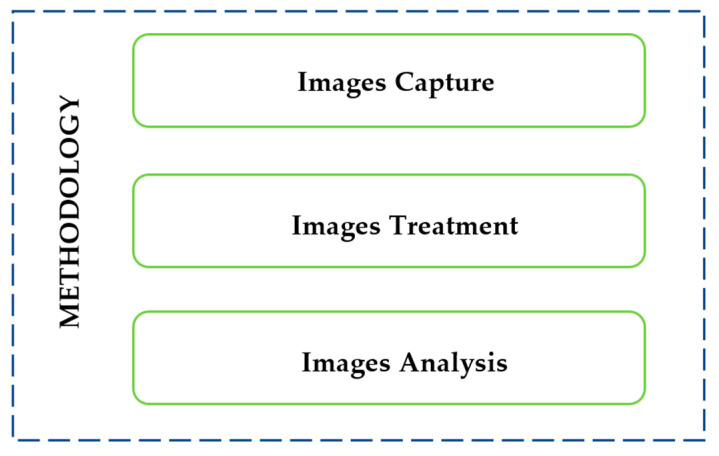
Methodology.

The methodology for quantifying the cracked areas of a PV panel was developed according to the IEC TS 60904-13 specification [37]. As shown in Figure 1, this methodology involves three phases: image capture, image treatment, and image analysis. This methodology is focused on systematically executing these phases in a manner that enables the analysis of numerous modules in a relatively efficient manner.

### 2.1. Image Capture

Image capture primarily involves four crucial aspects: the requirement of a dark environment, camera configuration and panel positioning, the characteristics of DC electric power supply, and the specifications of the EL camera.

According to [37], a darkened environment is favored for high-quality images. Thus, in this case, the EL-images of the panel were taken in a dark room whose inside conditions were suitable for the test.

**Figure 2:**
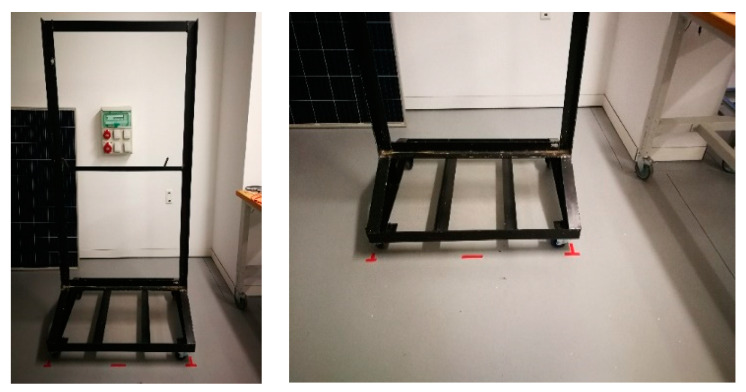
Workbench.

A bench was constructed to provide support for the modules during the test (Figure 2). This bench comprised a wheeled base for mobility and a vertical frame. The wheels were equipped with a locking mechanism to secure the position.

The camera used was completely suitable, since it covered the emission of the entire Si spectrum with high quantum efficiency, although its economic cost was higher, and the sensors of these cameras were made of InGaAs. This type of sensor is blind to visible light, so the detector behaves as a filter for ambient light [44]. The camera followed the specifications marked in [37]. For good EL-image quality, the focus and aperture angle of the camera were adjusted so that the distance at which the module was placed was proportional to the aperture angle of the lens and the exposure time of the camera. The camera was placed on a tripod, ensuring that its position would be the same for all shots. Once these adjustments were made, the images could be taken.

During this phase of the methodology, the positioning of PV modules and the EL-camera is crucial. In this context, the distance between them should be consistent throughout the entire procedure. This ensures the capture of high-quality images and pixel information in all EL-images taken. The distance between them was guaranteed, as the positions of both the bench and the EL-camera were marked on the laboratory floor.

**Figure 3:**
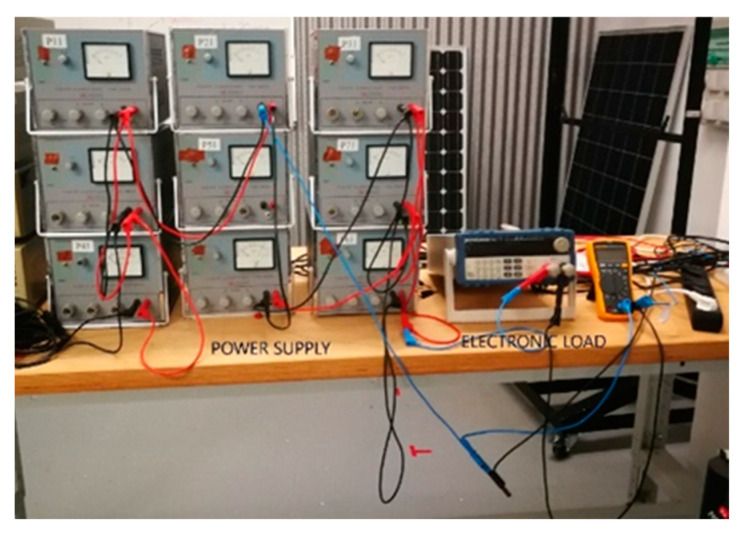
Power supply and electronic load connection.

**Figure 4:**
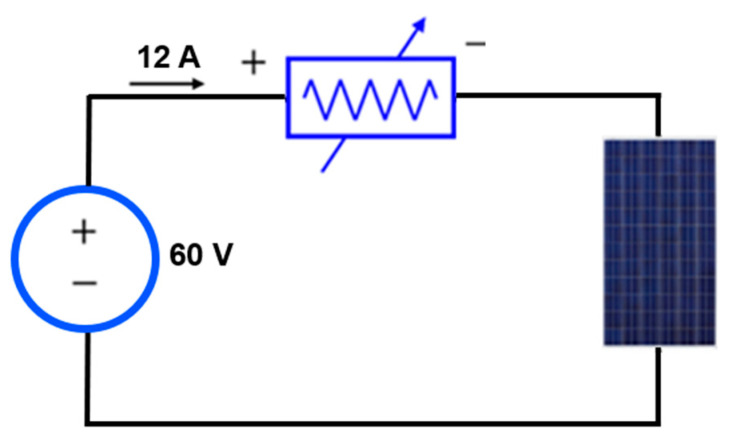
Setup for laboratory EL assay. Connection scheme.

For the power supply of the module during the EL-test, a set of DC power sources connected in series or parallel was used, alongside an electronic load that controlled the injected current into the PV module. The models of the DC power sources were Promax units, capable of providing a total current of 12 A at voltages up to 60 V, well beyond the requirements of the tests to be conducted. The electronic load model used herein was the SK Precision 8500, which was connected in series with the DC power sources. Figure 3 shows the connection between the DC power sources and the electronic load. Figure 4 provides the connection diagram for these components with the PV module. A voltmeter was employed to measure the voltage for each current polarization, as depicted in Figure 3.

**Figure 5:**
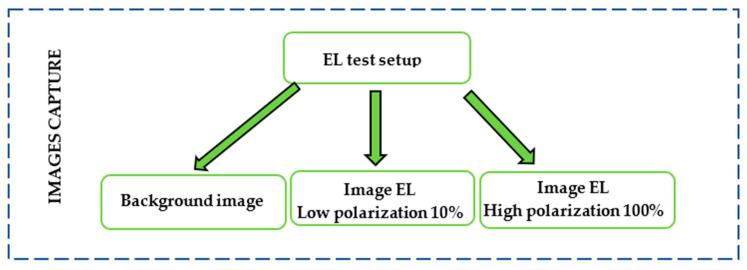
EL-image capture and setup procedure.

The image capture phase involved taking three images for each EL-test by injecting different polarizing currents through the PV panel under study. Figure 5 illustrates the image capture procedure, which consisted of taking three images: a background image, a low polarization-current image, and a high polarization-current image.

The first image, called the background image, was taken without injecting a current through the PV module. The purpose of this image was to establish the black color tone for areas of the module that did not generate electrical energy, such as electrical connection strips or junction lines between cells. These areas were referred to as inactive areas and were not taken into consideration when quantifying the percentage of cracked areas. Only the active areas were considered for calculations.

In the second image, a current equivalent to 10% of the short-circuit current (I_sc_) at standard test conditions (STC) was injected into the PV module. The images obtained with this polarization current exclusively revealed the areas damaged by type C cracks.

Finally, in the third image, a current equivalent to 100% of the I_sc_ at STC was injected into the PV module. Type B cracks exhibited dark regions in high-current EL-images; however, these dark areas vanished in low-current EL-images, indicating sustained electrical connection. Type C cracks occurred when certain areas became entirely electrically isolated from the circuit, resulting in their appearance as dark regions in EL-images under both low and high polarization currents [9]. Thus, the images obtained with high polarizing currents revealed all the damaged areas of the module, encompassing both type B and type C cracks.

Before obtaining the images and considering that dirt on the panel’s surface may have affected the results, it was necessary to clean it before capturing EL images. Then, after capturing the images, the results could be analyzed in various ways. In this study, the analysis included the assessment of cracked area percentages by module, substring, and cell. In the case of the cell, only the most affected cell within each substring was analyzed.

### 2.2. Images Procesing

For each EL-test, the processing was performed by utilizing information captured from the three EL-images. This study focused on types C cracks due to their potential impact on the performance of a PV module. In the case of type C cracks, damaged areas persisted in EL-images for both low- and high-polarized PV modules.

**Figure 6:**
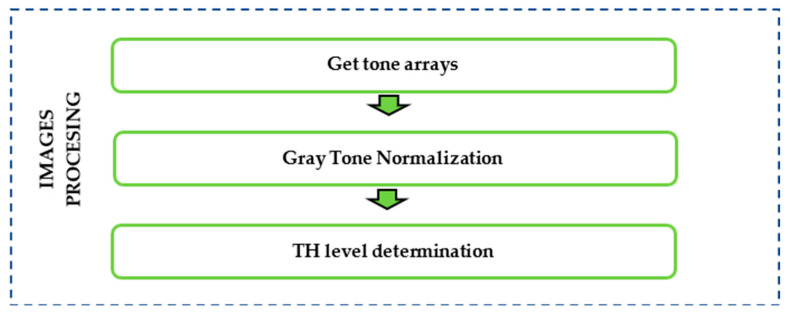
Procedure for image processing.

All information regarding damaged areas was derived from the captured images, underscoring the vital importance of image quality. Upon capturing EL-images, the sequential image processing steps for image treatment are depicted in Figure 6. As illustrated in this figure, a total of three steps were necessary to quantify the areas impacted by cracks.

In the next point, the approach taken in each step of the image processing will be further described.

#### 2.2.1. Obtaining Tone Matrices

**Figure 7:**
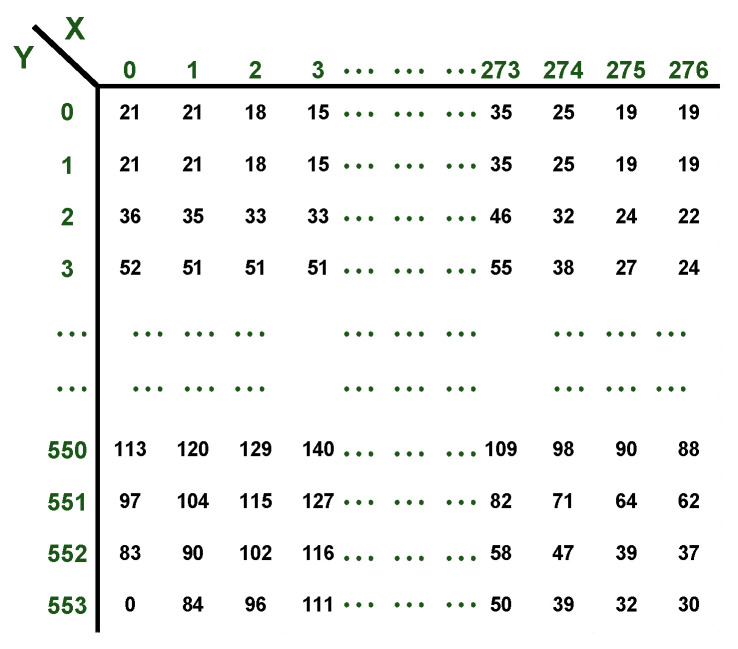
Grayscale matrix example.

After capturing the EL-images, analysis was conducted based on their pixel tones. The EL-images were captured in grayscale and saved in .png format. Then, corrections were applied to achieve, characterize, and optimize the desired image quality. Two image corrections were executed: frame subtraction and the highlighting of active areas. The frame subtraction was carried out between the background image and the EL-images on a pixel-by-pixel basis. The active zones were emphasized through pre-processing of the EL image to distinctly identify the dark zones within it. Subsequently, a grayscale matrix of the EL-image and the preprocessed El-image was obtained using Matlab© R2021b. Each element within the matrix represented the grayscale value of each pixel, where 0 corresponded to black and 255 to white. An example of a grayscale matrix is depicted in Figure 7, which has dimensions of 277 × 554 pixels, corresponding to a matrix containing 277 × 554 elements.

#### 2.2.2. Normalization of Gray Tones

The normalization of pixel tones is considered essential for subsequent comparisons between the results of the analysis of the tested module. Therefore, rather than assuming a grayscale tone range from 0 to 255, it was assumed that the tones ranged from a value of 0, representing black, to a value of 1, representing white in the grayscale pixels.

To conduct a more thorough analysis of the results, the obtained data matrices were represented using histograms. In these histograms, the X-axis represented the normalized grayscale tone ranging from 0 to 1, *i*, and the Y-axis showed its probability of occurrence, *p*(*i*), according to the expression derived from [6]:

(1) $$p\left(i\right)=\frac{n_{i}}{n},\;\;\;\;\;\;\;\;\;\;0\leq i\;\leq L$$

where:

$n_{i}$ is the number of pixels that have the same shade of gray;
n represents the total number of pixels that make up the studied image;
i is the gray level of a pixel;
L is the total number of gray levels, normalized by the maximum level.

#### 2.2.3. Determination of the Threshold or TH Level

Among the available methods for determining the TH level for image binarization, the selected method in this case was the one proposed by Noboyuki Otsu, commonly referred to as the maximum inter-class variance method [45]. This method is internationally recognized and is recommended by [37]. Furthermore, other references, such as [46,47], highlight Otsu’s method as one of the most effective for image segmentation, consistently delivering robust results for binary images.

Once the histogram was constructed, there was a gray level or tone, which we called TH (threshold), that delimited that region from the rest of the histogram. That point, tone in reality, divided the general distribution of the histogram into two Gaussian sub-distributions of the Function (1):

(2) $$\omega_{1}\left(l\right)=\sum_{i=1}^{l}p\left(i\right)\;\;\;\;\;\;\;\;\;\;\;\;\;\;\;\;\;\;\omega_{2}\left(l\right)=\sum_{i=l+1}^{L}p\left(i\right)$$

where *l* is the tested level and *L* is the total number of tones existing in the image and corresponding histogram. The means of each of the two sub-distributions were:

(3) $$\mu_{1}\left(l\right)=\sum_{i=1}^{l}\frac{i\cdot p\left(i\right)}{\omega_{1}\left(l\right)}\;\;\;\;\;\;\;\;\;\;\;\;\;\;\;\;\;\;\;\;\;\;\mu_{2}\left(l\right)=\sum_{i=l+1}^{L}\frac{i\cdot p\left(i\right)}{\omega_{2}\left(l\right)}$$

The average intensity between both distributions was:

(4) $$\mu_{T}=\omega_{1}\cdot \mu_{1}+\omega_{2}\cdot \mu_{2}$$

Otsu proposed an expression of the combined variance between both sub-distributions:

(5) $$\sigma^{2}\left(l\right)=\omega_{1}\left(l\right)\cdot {\left(\mu_{1}\left(l\right)-\mu_{T}\right)}^{2}+\omega_{2}\left(l\right)\cdot {\left(\mu_{2}\left(l\right)-\mu_{T}\right)}^{2}$$

The value sought for the TH level was that value of *l* that maximized the combined variance, that is:

(6) $$TH=\underset{l}{\max}\left\{\sigma^{2}\left(l\right)\right\}$$

### 2.3. Images Analysis

**Figure 8:**
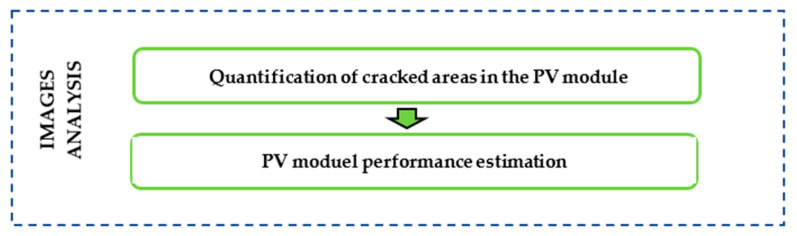
Procedure for image analysis.

After processing the EL-images, the analysis was conducted to quantify the darkest areas in the PV module, estimate the panel’s peak power, and ultimately determine the power loss and performance of the PV panel. The sequential steps for images anañysis are depicted in Figure 8.

#### 2.3.1. Quantification of Cracked Areas in the PV Module

Once the TH value had been determined, it was possible to identify the pixels that defined areas with cracks and the pixels that defined active areas. Thus, after calculating the TH, the grayscale matrix transformed into a resulting matrix of 0 s and 1 s, where the value 0 indicated cracks and the value 1 denoted active zones. By relating the number of pixels in each area to the total pixels in the image, the percentage of the study area affected by cracks was determined.

#### 2.3.2. Performance Analysis

The aim of this part of the procedure was to assess the performance of the analyzed PV module. According to [41], the power output correlates directly with the extent of cracking in the PV module. Thus, once the percentages of surface area affected by cracks had been identified, the performance of the PV panel could be calculated using the following expression:

(7) $$\eta =\frac{P_{STC}-P_{est}}{P_{STC}}$$

where:

$P_{STC}$ refers to the maximum power output under standard test conditions (STC), and $P_{est}$ represents the estimated maximum power output of the tested panel, calculated as follows:

(8) $$P_{est}=A\cdot I_{max(STC)}{\cdot U}_{max(STC)}$$

where A denotes the active PV area percentage within the panel. Depending on the desired analysis of the PV panel, Equation (8) can be modified to obtain P_est_. Subsequently, in Section 3, this expression is adapted to obtain P_est_ in cases where the panel was studied at the substring and PV cell levels.

Once the performance analysis result had been obtained, to verify the achieved outcomes, the module’s IV curve was measured. For this purpose, the PVPM1000x model of the PV curve tracer was employed. This device enables the determination of various PV panel parameters, encompassing maximum power (P_max_), short-circuit current (I_sc_), maximum power point current (I_pmax_), open-circuit voltage (V_oc_), maximum power point voltage (U_pmax_), and the fill factor (FF). Furthermore, to compute the STC parameters using the collected data, module temperature and irradiance were recorded. The IV tracer itself facilitated the computation of these STC parameters.

### 2.4. Implementation of the Methodology in Spreadsheet Software

The previously described methodology offers the advantage of being easily integrated into spreadsheet software such as Microsoft Excel© 365, which is widely adopted at an industrial level. Thus, the quantification of cracks in photovoltaic solar panels could be carried out in a quick and convenient manner, allowing for conclusions to be drawn by simply introducing the resulting matrix from the captured EL image.

**Figure 9:**
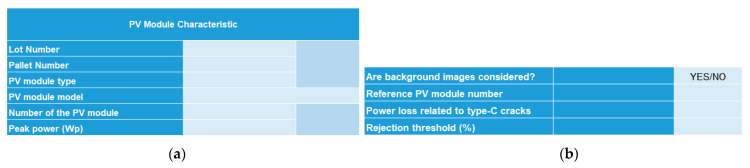
Image method. (**a**) General characteristics of the panels. (**b**) Data necessary for the calculation.

**Figure 10:**
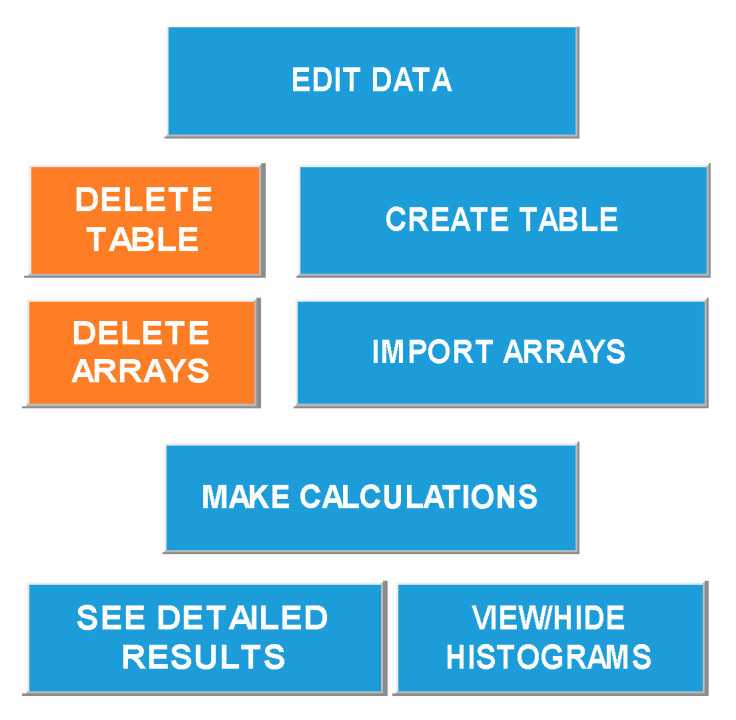
Method command buttons.

**Figure 11:**
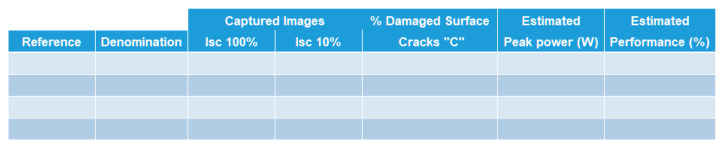
Table with results.

A spreadsheet software was designed to quantify type-C cracks and assess power loss using an EL-image following the methodology described in Section 2. To effectively use the spreadsheet software and navigate its interface, the following steps must be followed:

1. Enter the main parameters of the PV module: Provide logistics details, such as the number of PV modules and their type, model, and peak power. Figure 9a displays this section of the interface.
2. Choose technical considerations for applying the methodology: This involves determining whether background images should be considered, establishing power loss related to Type-C cracks, and setting a rejection threshold for PV panels based on their power loss, determining whether the panel is suitable for energy production in the PV plant. Figure 9b shows this section of the interface.
3. Validate inputs from steps 1 and 2: Click the “Create a Table” button located on the main tab of the method (Figure 10) to generate a table. This table will appear within the method, displaying results once the calculation is completed. The table will list all the panels to be analyzed. Figure 11 shows an example of a generated table.
4. Input EL-images into Matlab©: Introduce captured EL-images into Matlab© to obtain their corresponding resulting matrices. The incorporation of these matrices in the spreadsheet software will be accomplished by means of the “Import arrays” button, also located in the main tab (Figure 10). A matrix for each panel must be introduced. For each panel, an Excel sheet is generated. Consequently, each matrix is processed and analyzed separately.
5. Perform calculations: Click the “Make Calculation” button to swiftly obtain histograms and calculated values.

### 2.5. Application of the Methodology

The methodology was employed to calculate the percentage of cracks in a photovoltaic panel that has undergone multiple impacts on its back layer, analyzing its condition after each impact. This evaluation enabled a more detailed understanding of the evolution of the cracks throughout the various impacts, along with an estimation of the associated power loss. This research was performed inside a laboratory of the School of Engineering of the University of Cadiz, which met the suitable conditions for this work. A single PV module was used in the tests. The characteristics of the tested PV module are shown in Table 1. The PV module comprised three substrings, each with 24 cells connected in series. Each substring was equipped with a bypass diode for protection. Substring one encompassed the first two columns of cells, substring two covered the third and fourth columns of cells, and substring three included the last two columns.

**Figure 12:**
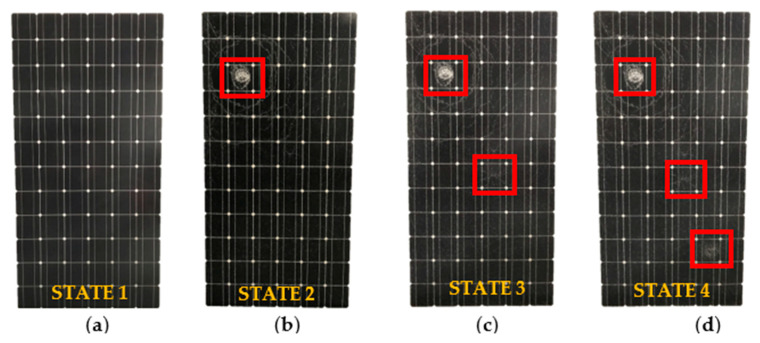
(**a**) Undamaged module. (**b**) Module with one damaged surface. (**c**) Module with two damaged surfaces. (**d**) Module with three damaged surfaces.

At the beginning, the PV module was in good condition, with no evident failures, and its production output remained within expected norms. To conduct the analysis on the impact of cracking on its performance, the module was subjected to incremental damage by applying several impacts to the rear of the module. The module was exposed to a total of three impacts, leading to the emergence of four distinct degradation states. Figure 12 depicts these four degradation states, which are enumerated as follows:

- States 1: PV panel in good condition. No failures or cracks were found during the initial visual inspection. A picture of this status is shown in Figure 12a.
- States 2: The PV panel received an impact on the B3 cell, leading to the identification of a cracked area within substring one through visual inspection. The impact is highlighted with a red square in Figure 12b.
- States 3: The PV panel received a second impact on the D7 cell, leading to the identification of a cracked area within substring two through visual inspection. The impacts are marked with green squares in Figure 12c.
- States 4: The PV panel received a third impact on the E10 cell, leading to the identification of a cracked area within substring three through visual inspection. The impacts are marked with yellow squares in Figure 12d.

To assess and quantify the cracked areas in the various degradation states, the developed methodology was applied to each of them. Thus, in each state, the performance estimation was made by examining the cracked areas across the entire panel, within each of the substrings, and in the most damaged PV cells within each substring.

## 3. Results

Once the test had been completely carried out, a total of 12 EL-images were taken. The EL-images corresponded to the background, low polarization at 10% of *I_sc_*, and high polarization at 100% of *I_sc_* for each state.

**Figure 13:**
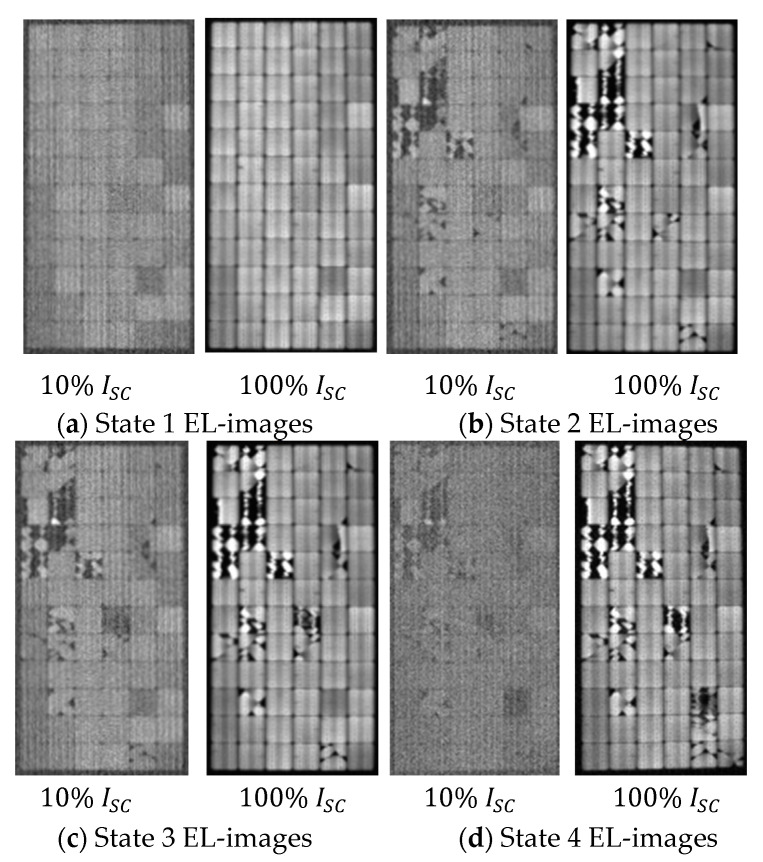
EL-images of each PV module state: (**a**) State 1; (**b**) State 2; (**c**) State 3; (**d**) State 4.

Figure 13 displays the EL-images obtained for each state. Background images are not included in this figure since they do not provide any visual information. Figure 13a depicts the EL-image of the panel in the initial state, displaying an absence of PV cracking that reflects its good status. Subsequent EL-images in Figure 13 illustrate a progressive increase in cracking levels as the panel transitioned through different states.

**Figure 14:**
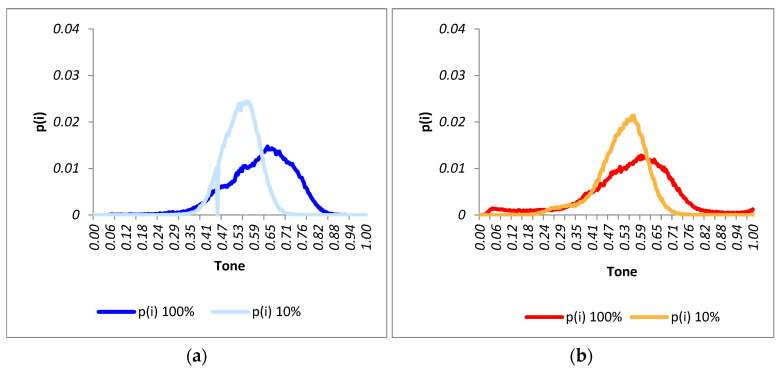

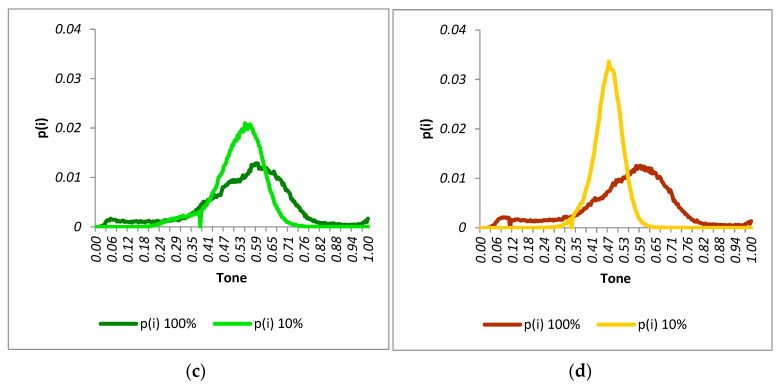
(**a**) PV histograms (state 1). (**b**) PV histograms (state 2). (**c**) PV histograms (state 3). (**d**) PV histograms (state 4).

The histograms obtained for each of the states at both polarized currents are shown in Figure 14.

Based on the resulting histograms, the following points are highlighted:

- For the undamaged module (state 1), the distribution exhibited exponentially bounded tails. In this scenario, the histogram’s shape closely resembled that of a normal distribution, with a median value of 0.44.
- The histogram’s shape in state 1 differed significantly from those obtained for states 2, 3, and 4. However, outside the tail areas, the histograms of states 2, 3, and 4 exhibited considerable similarity, indicating that the initial impact had a greater effect on the panel.
- The histograms for states 2, 3, and 4 demonstrated a notable shift towards darker tones in comparison to that of state 1. This shift appears logical due to the increased presence of areas with higher levels of cracking.
- The left tail of the distribution became heavier with the emergence of cracked areas, a phenomenon also documented in [48] that indicates the inactive areas of the module. The distribution related to state 4 stands out as the most significant in this aspect.
- While not to the same extent as the left tails, the right tails also exhibited an increase in states 2, 3, and 4 compared to state 1. This phenomenon occurred because of the glazing material formed around the dark areas, causing increased brightness upon excitation.

**Figure 15:**
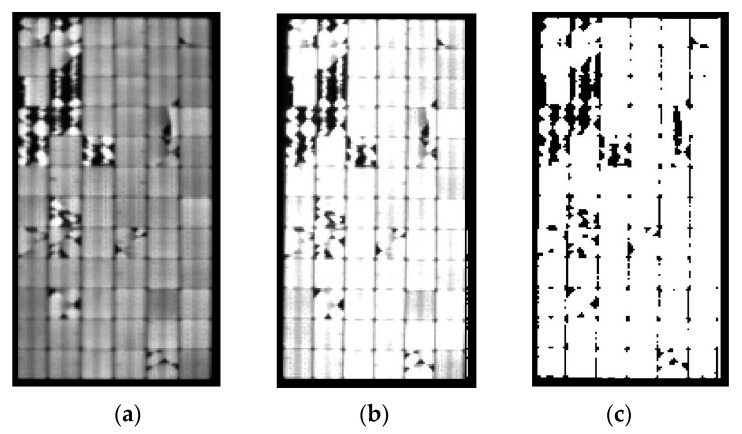
EL-images at different steps of the procedure for state 2. (**a**) EL-image. (**b**) Preprocessed EL-image. (**c**) Processed EL-image.

Figure 15 shows the EL-image at different steps of the procedure for state 2. The EL image captured by the camera is depicted in (a); the preprocessed EL image is shown in (b); and, finally, the processed EL image based on the obtained level of THD can be observed in (c).

Table 2 shows the results obtained for each state. This table contains the TH value, the percentage of Type C cracks, the P_est_ in STC, the power loss percentage for each state, and the estimated performance. The resulting percentage of type C cracks was based on the total surface area of the panel. The power loss was compared with the power established in the manufacturer’s datasheet.

In state 1, the obtained P_est_ was 194.56 Wp, representing a power loss of 0.23% and a performance of 99.77%. In state 2, the achieved P_est_ stood at 180.28 Wp, reflecting a power loss of 7.55% and a performance of 92.45%. In state 3, the obtained P_est_ was 177.78 Wp, resulting in a power loss of 8.83% and a performance of 91.17% respectively. Finally, in state 4, the P_est_ stood at 174.59 Wp, indicating a respective power loss of 10.47% and a performance of 89.53%.

**Figure 16:**
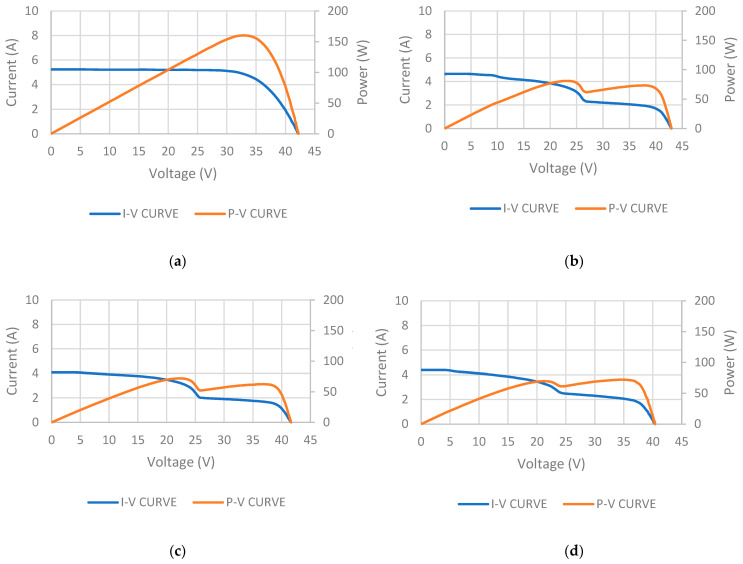
(**a**) I-V, P-V characteristic curve in state 1. (**b**) I-V, P-V characteristic curve in state 2. (**c**) I-V, P-V characteristic curve in state 3. (**d**) I-V, P-V characteristic curve in state 4.

For validation purposes, the P_est_ for each state was compared with the power measured by the I–V tracer. This test facilitated the acquisition of I-V and P-V curves for each state. Figure 16 illustrates the resultant I-V and P-V curves obtained for each state.

In Figure 16a, the test results for the I-V and P-V curves of state 1, conducted under an irradiance of 934 W/m^2^, are presented. The peak power achieved under STC was 180.2 W, indicating a power loss of 7.63% in comparison to the manufacturer’s datasheet.

In Figure 16b, the test results for the I-V and P-V curves of state 2, conducted under an irradiance of 896 W/m^2^, are presented. The peak power achieved under STC was 85.5 W, indicating a power loss of 56.2% in comparison to the manufacturer’s datasheet.

In Figure 16c, the test results for the I-V and P-V curves of state 3, conducted under an irradiance of 839 W/m^2^, are presented. The peak power achieved under STC was 79.0 W, indicating a power loss of 59.5% in comparison to the manufacturer’s datasheet.

In Figure 16d, the test results for the I-V and P-V curves of state 4, conducted under an irradiance of 961 W/m^2^, are presented. The peak power achieved under STC was 68.00 W, indicating a power loss of 65.14% in comparison to the manufacturer’s datasheet.

Table 3 presents a summary of the measured results. This table illustrates both the peak power (P) measured in STC and the corresponding power losses for each state according to the manufacturer’s datasheet.

Table 4 illustrates a comparison between the measured and estimated results for each state. A notable discrepancy between the estimated and measured values is evident, particularly in state 4, where there is a considerable difference of 141.47% in the estimated power compared to the measured power. This deviation arose due to the oversight of the internal connections within the PV panel. As the PV panel was segmented into three substrings (via bypass diodes), the direct proportionality between the areas affected by type C cracks and the resultant power loss was disrupted.

The analysis of the resulting I-V and P-V curves in Figure 16 reveals a similarity to those obtained when a PV panel is subjected to partial shadowing [49]. This suggests that the panel’s response to type C cracks may emulate behavior associated with experiencing partial shadowing, wherein a portion of the cell is rendered inactive, thus hindering energy production and its performance. Hence, the obtained results will be now analyzed based on substrings and individual cells.

### 3.1. Analysis by Substring

**Figure 17:**
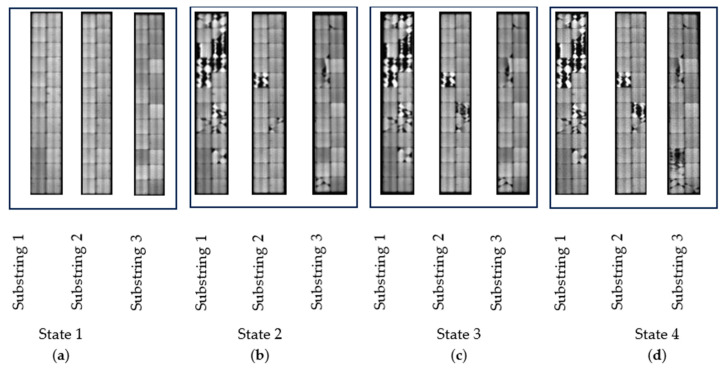
Module segmentation by substring corresponding to 100% *I_sc_*. (**a**) State 1. (**b**) State 2. (**c**) State 3. (**d**) State 4.

Here, the results obtained for each substring will be presented. Based on the resulting matrix derived from the entire PV module, the module’s results will be divided according to the number of substrings. In this case, the PV module is structured with three bypass diodes connected in parallel with 24 cells, thereby dividing the PV module into three parts. Figure 17 shows the EL-images obtained for each substring.

The estimated P of the panel was calculated considering the cracked area of each substring. As each substring was impacted by different cracked zones, the resulting I-V curve exhibited three maximum power points, one for each substring. Thus, based on Equation (9), the maximum power point for each substring (*S*) in the I-V curve was obtained as follows:

(9) $${P\left(S_{1}\right)}_{est}=A(S_{1})\cdot I_{max(STC)}\cdot \frac{U_{max(STC)}}{3}$$

(10) $${P\left(S_{2}\right)}_{est}=A(S_{2})\cdot I_{max(STC)}\cdot 2\frac{U_{max(STC)}}{3}$$

(11) $${P\left(S_{3}\right)}_{est}=A(S_{3})\cdot I_{max(STC)}\cdot \frac{U_{max(STC)}}{3}$$

where $A(S_{1})$, $A(S_{2})$ and $A\left(S_{3}\right)$ are the estimated cracked areas in substrings 1, 2, and 3, respectively, ordered according to the number of cracked areas, from least to greatest.

Consequently, the highest values among these three maximum power points indicate the estimated power output of the PV module.

(12) $$P_{est}=max\left\{{P\left(S_{1}\right)}_{est},{P\left(S_{2}\right)}_{est},{P\left(S_{3}\right)}_{est}\right\}$$

The results obtained in each state are shown in Table 5, where the area damaged by Type C cracks of each substring is shown, along with the corresponding power loss. In this case, the resulting percentage of type C cracks was based on the surface area of each respective substring. The power loss was calculated according to the manufacturer’s data sheet. Table 6 then illustrates a comparison between the measured and estimated results for each substring within each state.

According to the results shown in Table 6, a notable discrepancy between the estimated and measured values is particularly evident in state 4, showcasing a substantial difference of 126.25% between the estimated and measured power. This discrepancy emerged due to the influence of damage to a single PV cell, which was capable of affecting the behavior of the entire substring and thereby impacting the overall power output of the PV module. This observation, again, derives from the similarity between the effect caused by cracked inactive areas and partial shadowing [49].

### 3.2. PV Cell Analysis

**Figure 18:**
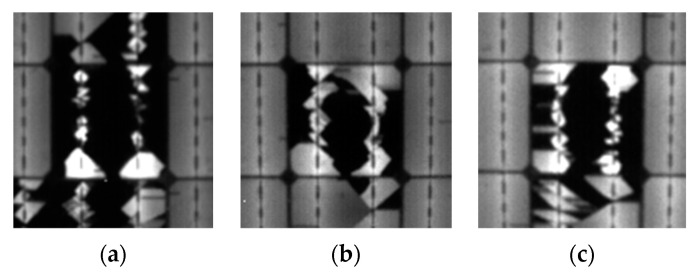
The most damaged photovoltaic cells in each string (State 4). (**a**) Substring 1: PV cell C5. (**b**) Substring 2: PV cell D7. (**c**) Substring 3: PV cell E10.

Following the observation taken in point 3.2, to conduct the cell-level analysis, the most damaged cells within each substring were considered. The obtained results are presented in Table 7, showing the damaged area with Type C cracks in each studied cell, along with their respective power losses. In this case, the resulting percentage of type C cracks was based on the surface area of each individual cell, respectively. Figure 18 displays the PV cells that incurred the most damage at the end of the test for each substring. In this instance, the most damaged cells for substring 1, 2, and 3 were B3, D7, and E10, respectively.

The P_est_ of the panel was calculated considering that the cracked area of the most damaged PV cell would affect the entire substring. As each substring was impacted by various cracked zones in a PV cell, the resulting I-V curve exhibited three maximum power points, one for each substring. In this case, the procedure to estimate the power was similar to the one used in the substring case. The difference is that, instead of considering the cracked area (*A*) as the cracked area of the whole substring, the considered cracked area was that of the PV cell most affected by the crack. Thus, the maximum power point for each substring (*S*) in the I-V curve was obtained as follows:

(13) $${P\left(S_{1}\right)}_{est}=A(C_{1})\cdot I_{max(STC)}\cdot \frac{U_{max(STC)}}{3}$$

(14) $${P\left(S_{2}\right)}_{est}=A(C_{2})\cdot I_{max(STC)}\cdot 2\frac{U_{max(STC)}}{3}$$

(15) $${P\left(S_{3}\right)}_{est}=A(C_{3})\cdot I_{max(STC)}\cdot U_{max(STC)}$$

where $A(C_{1})$, $A\left(C_{2}\right),$ and $A\left(C_{3}\right)$ represent the estimated cracked areas of the most affected PV cells in substrings 1, 2, and 3, respectively, ordered according to the number of cracked areas from least to greatest.

Consequently, the highest values among these three maximum power points indicate the estimated power output of the PV module.

(16) $$P_{est}=max\left\{{P\left(S_{1}\right)}_{est},{P\left(S_{2}\right)}_{est},{P\left(S_{3}\right)}_{est}\right\}$$

Table 8 presents a comparative analysis between the measured and P_est_ for the PV-cell analysis within each state. Based on the findings from this case, the P_est_ closely aligns with the measured values. These results affirm the similarity in the behavior of PV panels when affected by cracked or shadowed areas. In conclusion, if estimating the power output of a photovoltaic panel using an EL-image, it is crucial to consider the cell within each substring that is most affected by the cracks.

## 4. Case Study: Application in a Real Scenario

Previously, a test was conducted to estimate the power output of a panel that had received various impacts on its back layer, analyzing its condition after each hit. As a result, it was concluded that the most damaged PV cell in each string would be the primary factor affecting PV production. In this regard, when assessing whether a panel should be replaced through corrective maintenance actions, it is more influential for the same percentage of cracks to be concentrated in a single cell than if they are scattered throughout the substring or the panel itself.

To confirm this conclusion in panels that had been in operation in a real scenario, four different photovoltaic panels installed in a solar power plant were examined. The characteristics of the photovoltaic panels are shown in Table 9.

**Figure 19:**
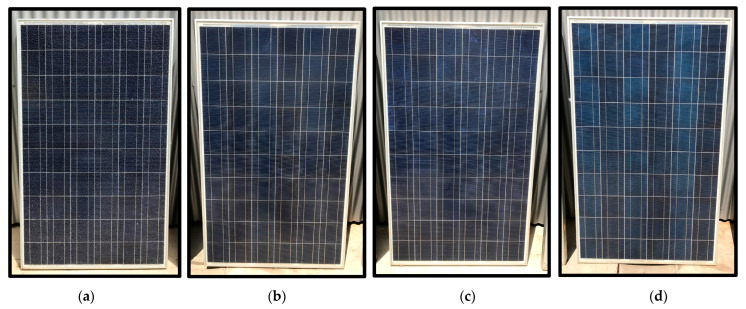
(**a**) PV module 1. (**b**) PV module 2. (**c**) PV module 3. (**d**) PV module 4.

In Figure 19, an image of each of the four modules is presented. Upon visual inspection, it was observed that module PV 1 had a broken front glass, resulting in the presence of cracks. The rest of the modules did not appear to have any anomalies in this regard, so no cracks were expected within them.

**Figure 20:**
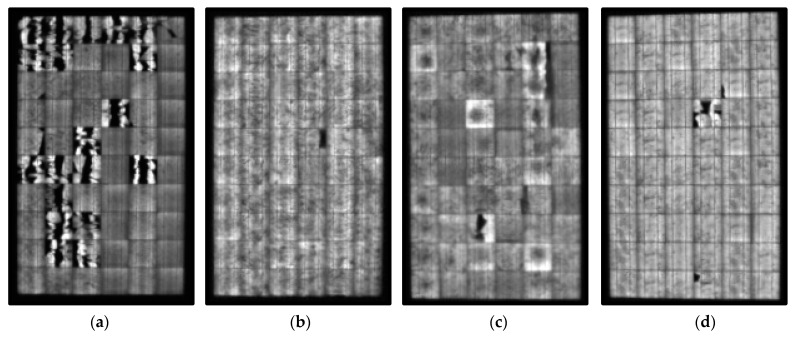
(**a**) PV module 1 EL-image. (**b**) PV module 2 EL-image. (**c**) PV module 3 EL-image. (**d**) PV module 4 EL-image.

Figure 20 displays the results obtained after conducting the EL test on each of the panels. As expected, Panel 1 showed a considerable amount of cracks. For Panels 2, 3, and 4, different cracks were observed, concentrated in various PV cells.

**Figure 21:**
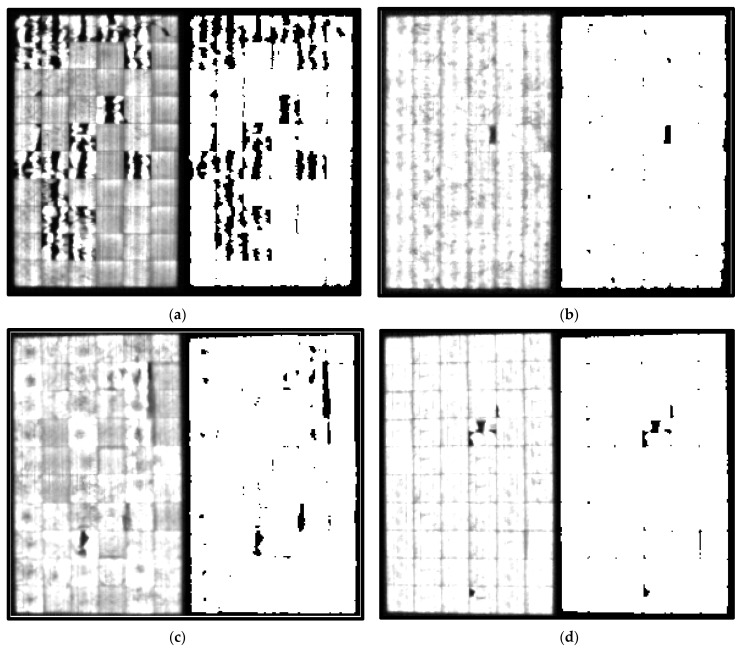
(**a**) Pre-processed and processed PV module 1 EL-image. (**b**) Pre-processed and processed PV module 2 EL-image. (**c**) Pre-processed and processed PV module 3 EL-image. (**d**) Pre-processed and processed PV module 4 EL-image.

Figure 21 displays the EL images once the developed methodology had been applied. For each PV module, both the pre-processed and processed images are presented.

In the processed images, it is possible to observe and quantify Type C cracks in each of the PV modules. The percentages of cracks recorded were 17.41% for PV module l 1, 2.64% for PV module 2, 3.24% for PV module 3, and 1.06% for PV module 4. However, based on the previously highlighted conclusion, evaluating the performance of the PV panel solely based on these crack percentages was deemed inadequate.

**Figure 22:**
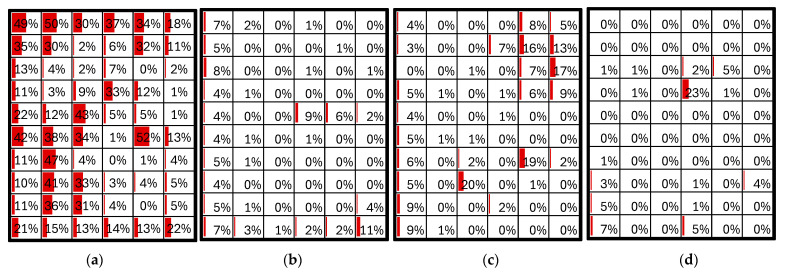
(**a**) Percentage of C-type cracks by PV cell in PV module 1. (**b**) Percentage of C-type cracks by PV cell in PV module 2. (**c**) Percentage of C-type cracks by PV cell in PV module 3. (**d**) Percentage of C-type cracks by PV cell in PV module 4. Each box represents a PV cell. The red color illustrates the percentage of cracks corresponding to each PV cell, enabling rapid comparison between them.

For a more precise assessment, it was proposed to analyze the crack percentage for each individual PV cell. Figure 22 provides the crack percentages for each cell in the panels, and these results were utilized to evaluate the performance of the PV panels. The obtained results are detailed in Table 10.

For validation purposes, the estimated power in each case was compared once again with the power measured by the curve tracer, revealing slight discrepancies between the estimated and measured outcomes. The results are shown in Table 11.

If, during corrective maintenance activities, a decision is needed regarding whether to replace a PV module, such a determination can be made by assessing the annual degradation level the module should exhibit. This analysis can be conducted by considering a linear degradation over time, following the approach employed in [22], where the average annual degradation rate of the module’s maximum power, as per the manufacturer’s warranty (WADR_Pmax_), is determined as:

(17) $${WADR}_{Pmax}=\frac{{WP}_{loss}}{w_{y}}$$

where ${WP}_{loss}\;$ is the power loss in % and $w_{y}$ represents the years of the module’s warranty.

The manufacturers guarantee a maximum PV module power loss during a certain time, usually 20% of the rated power during 20–25 years. Considering the most unfavorable scenario, i.e., a useful life of 20 years, the value of WAR would be 1%. Thus, considering this scenario and that the studied PV modules have been in production for 11 years, results exceeding an 11% degradation would surpass the anticipated degradation. However, since they would not reach the 20% degradation threshold, which marks the end of the panel’s useful life, replacement should not be considered.

Finally, Table 12 provides a qualitative summary of the actions to be taken during the corrective maintenance activities for the studied panels. In this summary, it is recommended to replace Panel 1, as it exhibited degradation beyond the acceptable threshold. Meanwhile, the remaining panels showed levels of degradation exceeding their anticipated limits, potentially indicating a premature end to their useful lives.

## 5. Conclusions

The primary functionality of the presented method revolves around EL-images of a photovoltaic (PV) panel. This testing method offers significant advantages over other assays, such as I-V curve measurement. Requiring darkness conditions, these analyses can be conducted at night without disrupting the plant’s production. This feature allows for these assays to be performed seamlessly, without interrupting the regular operation of the photovoltaic plant.

To practically conduct these tests, the development of a testing procedure would be necessary, incorporating the application of the current method. Hence, in scenarios wherein reduced generation in any part of the photovoltaic plant is observed, typically detected by its SCADA system, EL testing could be employed to assess whether certain panels are affected by cracked cells. This would enable the evaluation of the performance of the affected area. Presently, numerous studies are utilizing UAVs primarily for visible defect detection [50,51]. Once the affected panels are identified, they can be individually analyzed using the developed method without the need for dismantling, providing an on-field solution.

For future improvements in its implementation, a comprehensive evaluation of a string of PV panels could be considered to streamline maintenance tasks based on their impacts on production.

## 6. Discussion

Recognizing the significance of automatically studying the behavior of photovoltaic (PV) modules when impacted by cracked areas, this article introduces the development of a method. This method, designed in accordance with the IEC TS 60904-13 specification, demonstrates the capability to estimate the power loss of PV panels affected by cracked zones using EL-images.

The primary objective of this article, given the increasing size of power plants and their expanding footprint, was to devise a swift and straightforward method for assessing the location of cracks in PV panels. The aim was to enhance decision making for corrective maintenance actions. In this context, a method was developed within the realm of maintenance tasks that is capable of swiftly delivering results through the utilization of an EL image of a photovoltaic panel. The method underwent rigorous testing with a PV panel. To assess the influence of cracking on its performance, the module underwent incremental damage through several impacts applied to its rear surface. Three impacts in total were administered, resulting in the emergence of four distinct degradation states. For validation purposes, the estimated peak power for each state was cross-referenced with the power measured using an I-V tracer.

Upon analyzing the module’s performance across all states, it became evident that the module’s power loss did not exhibit direct proportionality to the percentage of cracks in the PV module. Interestingly, the panel’s behavior closely resembled instances of partial shading. As a result, estimating power loss necessitated calculating the percentage of cracks in the most affected cell within each substring. Consequently, this type of analysis should focus on evaluating the cracked zones of all cells comprising the panel, with the most affected cell in each substring potentially dictating the power at the maximum power point of the photovoltaic panel. Hence, we conclude that cell-level analysis stands as the most suitable method for estimating power output from surfaces damaged by C-type cracks. Based on the obtained results, the described methodology demonstrates the ability to accurately estimate the power of an FV panel affected by type-C cells. In this case, for the PV panel utilized in the current study, which concluded with a total crack percentage of 10.47%, the estimated power loss amounted to 62.92%. This loss stemmed from the concentration of C-type cracks within a single PV cell.

Subsequently, to exemplify its application in maintenance tasks, the developed methodology was employed to assess the conditions of four photovoltaic panels installed in a solar power plant. The obtained results informed decisions on whether the panels should be replaced or could still be deemed useful. In this specific case, it was determined that one of the panels should be replaced, while the others, despite exhibiting small percentages of cracks in the panel, demonstrated advanced states of degradation, as the few cracks were concentrated in specific cells.

The presented method concentrates on utilizing EL-images in PV panels, presenting advantages over other testing methodologies by enabling nighttime analysis without disrupting plant production. It could be seamlessly integrated into a practical testing protocol to evaluate panel performance, especially in scenarios where diminished generation is observed. This approach, potentially employing UAVs, facilitates individual panel analysis without the need for dismantling, providing an efficient on-field solution. Future enhancements in procedures might involve a more comprehensive evaluation method to precisely assess the impact of affected panels on the overall system.

## Figures and Tables

**Table 1:** Technical characteristics of the PV.

Manufacturer	LUXOR (Stuttgart, Germany)
Solar Module	ECO LINE 72/185–195 W
PV Module type	LX-195M/125-72+
Electrical characteristics	195 W_p_ (+1.5/6.49 W_p_)
	U_mpp_ = 36.74 V, I_mpp_ = 5.31 A,
	I_sc_ 5.79 A, U_oc_ = 44.04 V,
Module dimensions	Area: 158 × 80.8 = 12,766 cm^2^

**Table 2:** PV module performance summary by module (designed method).

	TH	Surface Damaged by Type C Cracks (%)	P_est_ (Wp)	Power Loss (%)	Estimated Perfomance (%)
State 1	0.6412	0.23	194.56	0.23	99.77
State 2	0.6412	7.55	180.28	7.55	92.45
State 3	0.6314	8.83	177.78	8.83	91.17
State 4	0.6941	10.47	174.59	10.47	89.53

**Table 3:** Summary of the measured results for each state.

	P (Wp)	Power Loss (%)	Measured Performance (%)
State 1	180.21	7.59	92.41
State 2	85.51	56.15	43.85
State 3	79.00	59.49	40.51
State 4	72.30	62.92	37.08

**Table 4:** Comparative analysis of power losses between measured and estimated values for each state.

	Surface Damaged by Type C Cracks (%)	P_est_ in STC (Wp)	P Measured in STC (Wp)	Difference (%)
State 1	0.23	194.56	180.21	+7.97
State 2	7.55	180.28	85.51	+110.86
State 3	8.83	177.78	79.00	+125.04
State 4	10.47	174.59	72.30	+141.47

**Table 5:** PV module performance summary by substring.

	TH_100_	Surface Damaged by Type C Cracks (%)	P_est_ (W)	Power Loss (%)	Estimated Performance (%)
State 1					
Substring 1	0.6412	0.22	194.49	0.26	99.74
Substring 2	0.6412	0.15			
Substring 3	0.6412	0.31			
State 2					
Substring 1	0.6412	15.46	164.93	15.42	84.58
Substring 2	0.6412	3.47			
Substring 3	0.6412	3.93			
State 3					
Substring 1	0.6314	16.15	163.58	16.11	83.89
Substring 2	0.6314	5.30			
Substring 3	0.6314	5.12			
State 4					
Substring 1	0.6941	16.15	163.58	16.11	83.89
Substring 2	0.6941	5.53			
Substring 3	0.6941	9.68			

**Table 6:** Measured and estimated results per substring.

	P_est_ (Wp)	P Measured (Wp)	Difference (%)
State 1	194.49	180.21	+7.93
State 2	164.93	85.51	+92.87
State 3	163.58	79.00	+107.06
State 4	163.58	72.30	+126.25

**Table 7:** PV module performance summary by PV cell.

	PV Cell	TH_100_	Type C Cracks (%)	P_est_ (Wp)	Power Loss (%)	Estimated Performance (%)
State 1						
Substring 1	A12	0.6412	1.67	188.99	3.08	96.92
Substring 2	D1	0.6412	0.83			
Substring 3	F12	0.6412	3.13			
State 2						
Substring 1	B3	0.6412	66.67	75.19	61.44	38.56
Substring 2	C5	0.6412	42.19			
Substring 3	E4	0.6412	25.83			
State 3						
Substring 1	B3	0.6314	67.11	72.07	63.04	36.96
Substring 2	D7	0.6314	44.58			
Substring 3	E4	0.6314	27.68			
State 4						
Substring 1	B3	0.6941	68.44	61.56	68.43	31.57
Substring 2	D7	0.6941	46.27			
Substring 3	E10	0.6941	60.27			

**Table 8:** Measured and estimated results per cell.

	P_est_ (Wp)	P Measured (Wp)	Difference (%)
State 1	188.99	180.21	+4.88
State 2	75.19	85.51	−12.07
State 3	72.07	79.00	−8.77
State 4	61.56	72.30	−14.85

**Table 9:** Technical characteristics of the power plant PV panels.

	PV Module 1	PV Module 2	PV Module 3	PV Module 4
Manufacturer	TynSolar	TynSolar	TynSolar	TynSolar
Model	TYN-220P6 220Wp	TYN-220P6 205Wp	TYN-220P6 205Wp	TYN-220P6 220Wp
Electrical Characteristic	P = 220 W_p_	P = 205 W_p_	P = 205 W_p_	P = 220 W_p_
	U_mpp_ = 30.12 V	U_mpp_ = 29.58 V	U_mpp_ = 29.58 V	U_mpp_ = 30.12 V
	I_mpp_ = 7.30 A	I_mpp_ = 6.93 A	I_mpp_ = 6.93 A	I_mpp_ = 7.30 A
	I_sc_ = 7.95 A U_oc_ = 36.06 V	I_sc_ = 7.47 A U_oc_ = 35.94 V	I_sc_ = 7.47 A U_oc_ = 35.94 V	I_sc_ = 7.95 A U_oc_ = 36.06 V

**Table 10:** Summary of the performance of the PV modules extracted from the PV solar plant by PV cell.

	PV Cell	TH_100_	Type C Cracks (%)	P_est_ (Wp)	Power Loss (%)	Estimated Perfomance (%)
PV module 1						
Substring 1	A1	0.4647	49.62%	104.49	46.42	47.49
Substring 2	C5	0.4647	42.56%			
Substring 3	E6	0.4647	52.48%			
PV module 2						
Substring 1	A3	0.5471	8.30%	181.70	6.82	88.63
Substring 2	D5	0.5471	9.30%			
Substring 3	F10	0.5471	11.36%			
PV module 3						
Substring 1	A10	0.5745	9.24%	163.91	15.95	79.95
Substring 2	C8	0.5745	20.04%			
Substring 3	E7	0.5745	18.80%			
PV module 4						
Substring 1	A10	0.6412	7.25%	169.00	13.34	76.82
Substring 2	D4	0.6412	23.14%			
Substring 3	F8	0.6412	4.55%			

**Table 11:** Comparison between measured and estimated results.

	P_est_ (Wp)	P Measured (Wp)	Difference (%)
PV module 1	104.49	102.70	0.81
PV module 2	181.70	181.28	0.20
PV module 3	163.91	175.41	−5.61
PV module 4	169.00	170.35	−0.62

**Table 12:** Qualitative summary of the actions to be taken.

PV Module	Visual Inspection	EL-Imagen	Percentage of Cracks in the Whole Module	Most Affected PV Cell by C-Type Cracks	Estimated Power Loss	Decision Making
PV module 1	Broken front glass	A large number of cracks scattered across the entire panel.	17.41%	52.48%	46.42%	Replace
PV module 2	No visible cracks	A small number of cracks, but concentrated in specific PV cells.	2.64%	11.36%	6.82%	Not to replace, but to monitor in the upcoming years.
PV module 3	No visible cracks	A small number of cracks, but concentrated in specific PV cells.	3.24%	18.80%	15.95%	Not to replace, but to monitor in the upcoming years. Premature end of useful life.
PV module 4	No visible cracks	A small number of cracks, but concentrated in specific PV cells.	1.06%	23.14%	13.34%	Not to replace, but to monitor in the upcoming years. Premature end of useful life.

## Data Availability

The data presented in this study are available upon request from the corresponding author.
